# The evolution of dual meat and milk cattle husbandry in Linearbandkeramik societies

**DOI:** 10.1098/rspb.2017.0905

**Published:** 2017-08-02

**Authors:** Rosalind E. Gillis, Lenka Kovačiková, Stéphanie Bréhard, Emilie Guthmann, Ivana Vostrovská, Hana Nohálová, Rose-Marie Arbogast, László Domboróczki, Joachim Pechtl, Alexander Anders, Arkadiusz Marciniak, Anne Tresset, Jean-Denis Vigne

**Affiliations:** 1 Département Ecologie et Gestion de la Biodiversité, CNRS—Muséum National d'Histoire Naturelle—Sorbonne Universités, Archéozoologie, Archéobotanique: Sociétés, Pratiques et Environnement (UMR 7209), CP 56, 55 rue Buffon, 75005 Paris, France; 2 Institute for Prehistoric and Protohistoric Archaeology, Christian-Albrechts University, Kiel, Johanna-Mestorf Strasse 2-6, 24098 Kiel, Germany; 3 Laboratory of Archaeobotany and Palaeoecology, University of South Bohemia, Na Zlaté stoce 3, 37005 České Budějovice, Czech Republic; 4 ARCHIMEDE (UMR7044), Université de Strasbourg, Misha, 5 Allée du Général Rouvillois, 67083 Strasbourg Cedex, France; 5 Institute of Archaeology and Museology, Masaryk University, Arna Nováka 1, 602 00 Brno, Czech Republic; 6 István Dobó Castle Museum, 3300 Eger, Vár 1, Hungary; 7 Kelten Römer Museum Manching, Im Erlet 2, 85077 Manching, Germany; 8 HAS-ELTE Research Group for Interdisciplinary Studies, Múzeum Körút 4/B, 1088 Budapest, Hungary; 9 Instytut Archeologii UAM, Collegium Historicum, ul. Umultowska 89D, 61-614 Poznań, Poland

**Keywords:** Neolithic, husbandry practices, Linearbandkeramik, cattle, mortality profiles, milk

## Abstract

Cattle dominate archaeozoological assemblages from the north-central Europe between the sixth and fifth millennium BC and are frequently considered as exclusively used for their meat. Dairy products may have played a greater role than previously believed. Selective pressure on the lactase persistence mutation has been modelled to have begun between 6000 and 4000 years ago in central Europe. The discovery of milk lipids in late sixth millennium ceramic sieves in Poland may reflect an isolated regional peculiarity for cheese making or may signify more generalized milk exploitation in north-central Europe during the Early Neolithic. To investigate these issues, we analysed the mortality profiles based on age-at-death analysis of cattle tooth eruption, wear and replacement from 19 archaeological sites of the Linearbandkeramik (LBK) culture (sixth to fifth millennium BC). The results indicate that cattle husbandry was similar across time and space in the LBK culture with a degree of specialization for meat exploitation in some areas. Statistical comparison with reference age-at-death profiles indicate that mixed husbandry (milk and meat) was practised, with mature animals being kept. The analysis provides a unique insight into LBK cattle husbandry and how it evolved in later cultures in central and western Europe. It also opens a new perspective on how and why the Neolithic way of life developed through continental Europe and how dairy products became a part of the human diet.

## 1. Introduction

Milk production and consumption in prehistoric societies has been a centre of debate for decades [1–3], primarily owing to the majority of human populations being lactose intolerant after weaning, when the lactase enzyme production is switched off [4]. Consumption of milk products with high lactose content can cause extreme diarrhoea and gas [5]. Populations with the lactase persistence (LP) phenotype have been found to be strongly associated with dairy ruminants [5,6]. The mutation *−13.910T* has been found to be the cause of LP and is present in 95% of northern Europeans [7]. Selective pressure on LP has been modelled to have started between 6000 and 4000 BC in central Europe and spread to northwestern populations [8]. Although other studies suggest an alternative history [9], it is clear that the relationship between dairying and LP is complicated [10]. Indeed, it is possible to (i) develop lactose tolerance with gut flora, and (ii) by processing lactose is removed [11]. Archaeological evidence suggests that dairying may have been practised as soon as ruminants were domesticated [12], and appears to have played an important role in the spread of Neolithic way of life in some parts of the Mediterranean [13,14]. Furthermore, high milk lipid concentrations have been found in ceramic sherds strongly associated with high indices of cattle remains at seventh millennium BC sites in Northwest Anatolia [15]. Could dairying also have played a role in the spread of the Neolithic following the Danubian route (between the sixth and fifth millennium BC), where cattle were dominant? Does the discovery of pottery sieves with evidence of milk lipids in the late sixth millennium in Poland [16,17] reflect an isolated regional peculiarity for cheese making, or does it indicate a more generalized milk exploitation in north-central Europe during the early Neolithic? Here, we address these issues by studying cattle age-at-death data from 19 sites from the Linearbandkeramik (LBK) culture.

The LBK culture is believed to have originated in the Transdanubian region around 5600 cal. BC. The formative phase has been modelled to have begun 5545–5485 cal. BC (1*σ*) and ended 5420–5360 cal. BC (1*σ*) with a secondary expansion into the regions east, north and west of this area all settled by 5300 cal. BC [18]. Cattle were an important component of the LBK culture and may have contributed to its success and rapid expansion [19–23]. The study of LBK archaeozoological remains is hampered by acidic burial environments owing to the loess soils and our present understanding is incomplete [19]. However, the proportion of cattle bones (NISP) varies from 88.7% (Poland) to 43% (Baden-Württemberg) [20]. Previous studies of LBK cattle husbandry have proposed that meat was the main focus [19,20,24,25]. Bogucki [19,26] proposed a component of milk production primarily based on the presence of ceramic sieves, possibly used for cheese making. This was later confirmed by the presence of milk lipids [16]. More recently from the Czech Republic and France, there is growing evidence for dual exploitation of milk and meat using age-at-death analysis [27–29]. Maintaining slow-growing/maturing animals such as cattle would have represented an ‘investment’ [26,30]. This investment had major implications on the development of the symbolic role of cattle and social inequality [31]. Meat in a number of present-day societies is reserved for special occasions and ritual feasting events [32]. Whereas dairy husbandry in comparison would have less impact on small herds [30,33] and may have been more suitable for the establishment of LBK cattle herds.

Mortality data and profiles based on observations of dental eruption, wear and replacement stages can be used to study and characterize cattle slaughter management, which in turn can be used to study husbandry practices [34–36]. For the LBK area, these types of studies have so far been limited to regional studies [25,27–29]. Here, we investigate LBK cattle husbandry through statistical analyses of an age-at-death dataset based on 19 sites from across the LBK cultural zone. These analyses will help us to characterize LBK cattle husbandry on a global scale for the first time, and explore its evolution in subsequent cultural phases.

## 2. Material and methods

### (a) Material

**Figure 1.**
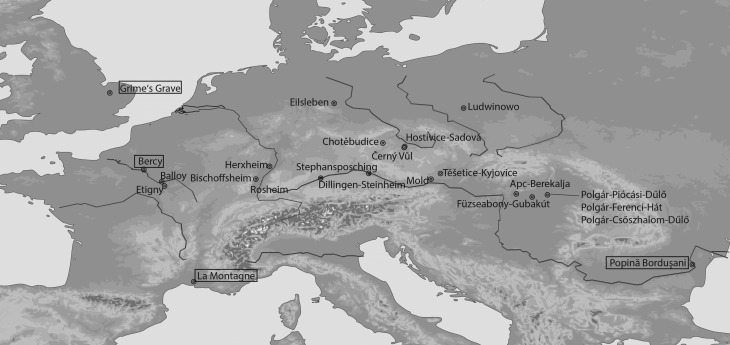
Map showing the locations of the sampled and reference (in boxes) sites.

The dataset represents 19 sites (27 contexts; total number of teeth = 1142) from central and northwestern Europe (figure 1; electronic supplementary material, S1). These sites date from 5600 to 4600 cal. BC. Four sites from Hungary represent the LBK. One of them belongs to the LBK in Transdanubia (TLP; Apc-Berekalja) and the remaining sites belong to the so-called Alföld Linear Pottery (ALP; Füzseabony-Gubakút, Polgár-Piócási-dűlő and Polgár-Ferenci-hát). The LBK phenomenon in Hungary was distributed mainly to the west of the Danube, whereas the ALP lies east of the Danube. This culture differs in pottery styles from TLP/LBK, although it has many similarities with TLP and LBK phenomenon [37]. Furthermore, the ALP and TLP cultures are dated to the Hungarian Middle Neolithic and follow the Starčevo (Transdanubia) and the Körös (Hungarian Plain). The remaining sites represent the typical LBK culture of central Europe dating from 5500 to 4900 BC. Eilsleben and Mold belong to initial phases of the LBK in their respective regions, with the majority of the sites belonging to the phases after the initial development of the LBK except for Herxheim and Etigny representing the final phases. Two sites were included to represent cultures that follow LBK: Polgár-Csőszhalom-dűlő (Hungarian Late Neolithic (HLN: 4840–4560 cal. BC)) and Balloy (Villeneuve St Germain (VSG: 4600–4450 cal. BC). These have been included to determine whether there were changes to cattle husbandry over time and if these changes are associated with the adoption/development of new ceramic cultures.

### (b) Age determination method

Dental wear stages [38] for the fourth lower deciduous pre-molar (D4), first molar (M1), second molar (M2) and third molar (M3) and crown height, DAP and DT [34] for the molars were recorded. M1 and M2 were differentiated using the distance anterior to posterior (DAP) and distance transverse (DT). Legge [35] age classes were used as they have been found to be more accurate [39]. Teeth were attributed to each age class following Legge [35]. Because of the fragmented nature of the remains and of the infrequent recovery of complete or large portions of mandibles, we included isolated teeth in the final count. Isolated teeth found to belong to several age classes were divided between them according to the respective time length of the age classes, according to Vigne [40].

### (c) Statistical analysis

The mortality profiles were based on number of teeth (*N*) and not minimum number of individuals, which is not linearly connected to sample size [41] and were generated using an R code described in [42] adapted for Legge [35] age classes to analyse cattle mortality (electronic supplementary material, S2 and S3). The published code in [42] being for caprines and is based on the Payne [43] age classes. Credible intervals for each age class were computed using the Dirichlet function (Dirichlet prior = 0.5; number of simulations = 2000).

The correspondence analysis (CA) of the mortality data was carried out using the Dirichlet function as described in [42] and adapted for Legge age classes [35]. The use of the Dirichlet function reduces the stochastic effect from small samples. The CA biplots can be used to elucidate trends within the data to generate hypotheses. We chose four reference profiles for milk and meat exploitation based on archaeological examples to aid the interpretation of the CA. These did not contribute to the overall inertia of the plot, so therefore did not affect the profile and age class distribution.

All analyses and biplots were produced using the free platform R program (v. 3.03.3 [44]) using R studio. R packages: ggplot2 (v. 1 [45]), grid (v. 3.0.2 [44]), gridExtra (v. 0.9.1 [46]), gtools (v. 3.4.2 [47]), ca (v. 0.55 [48]), LaplacesDemon (v. 16.0.1 [49]), MASS (v. 7.3-37 [50]) and PAST (v. 3.15) [51].

### (d) Reference profiles using archaeological examples

**Figure 2.**
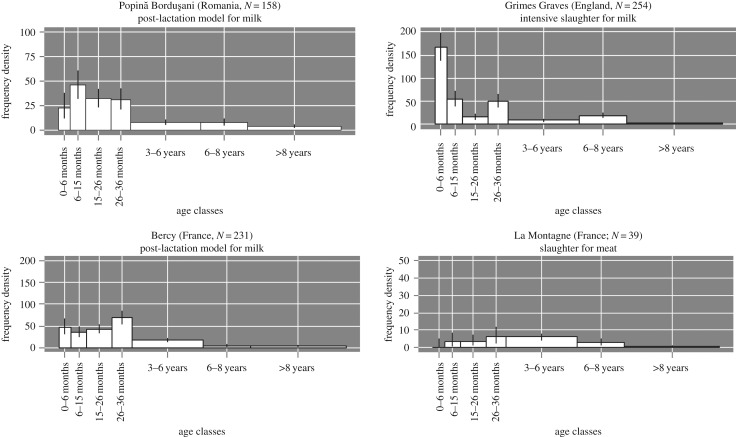
The four reference mortality profiles for post-lactation slaughter, intensive slaughter for milk and slaughter for meat production from the archaeological sites of Popină Borduşani (Romania), Bercy (France), Grimes Graves (England) and La Montagne (France), (*N*, number of teeth).

Identification of dairy husbandry from archaeozoological remains is difficult owing to unsuitable models based on modern herds, to poor recovery of archaeozoological material and inherent taphonomic bias [52] and to a lack of standard methodological techniques [36,53]. To overcome these issues, we have taken a standard methodological approach [35] coupled with a Bayesian statistical method [42] to combat stochasticity. Well-excavated sites were chosen with good preservation conditions. Application of ethnographic models to archaeological material is problematic because of the taphonomic and preservation biases. Therefore, we opted for mortality profiles from archaeological assemblages. We have chosen four sites as models for dairy and meat exploitation (electronic supplementary material, S1; figure 2).

The archaeological sites of Bercy (late fifth millennium BC, France) and Popină-Borduşani (late fifth millennium BC, Romania) were chosen as models of post-lactation slaughter. This has been proposed to be a model for dairy management in prehistoric herds, given that traditional cattle breeds do not release into the cistern without stimulation by suckling or the presence of the calf or a substitute [54]. In this model, milk is shared between the offspring and humans, and the calf is either kept or slaughtered at the end of lactation between six and 11 months. This decision depends on a number of parameters such as fodder resources and husbandry strategy. For both sites, bone collagen extracted from mandible remains were analysed for stable isotopes (*δ*^13^C and *δ*^15^N) to examine the transition from suckling to a post-weaning diet. The results proved that animals within the post-lactation slaughter peak were weaned [55,56], therefore providing indirect evidence that cattle were managed for milk. These profiles are characterized by relatively high slaughter in age class 6–15 months and may also represent a slaughter of young adults for meat production, for example, in age classes 15–26 and 26–36 months.

We have included the profile from Grimes Graves (Bronze Age, England) to explore intensive dairy husbandry. Legge [35] found at the site Grimes Graves, calves were culled less than six months old but aged greater than one month. He proposed this was evidence for an intensive slaughter associated with dairying and not natural mortality, which is highest in young animals prior to one month. Given that there is no other proxy for dairy production/processing at the site, this type of mortality could also be an indication of high natural mortality [52] as well as early slaughter associated with a shorter lactation length [57] or in response to extreme climate [57].

An auroch mortality profile from La Montagne (France, Mesolithic [58]) was chosen as a reference profile for meat exploitation. The largest peak is in age class 3–6 years, which suggests that hunters targeted animals at their prime weight, similarly to traditional meat exploitation [59]. By choosing a wild animal mortality profile, we can be sure that no animals were being slaughtered as a consequence of dairy husbandry. However, as a hunting profile is similar to a meat-focused one, there may be issues of equifinality, as the provision of meat by farmers may be a result of diverse management practices or treatment of domesticated herds as property.

## 3. Results

### (a) Mortality profiles

**Figure 3.**
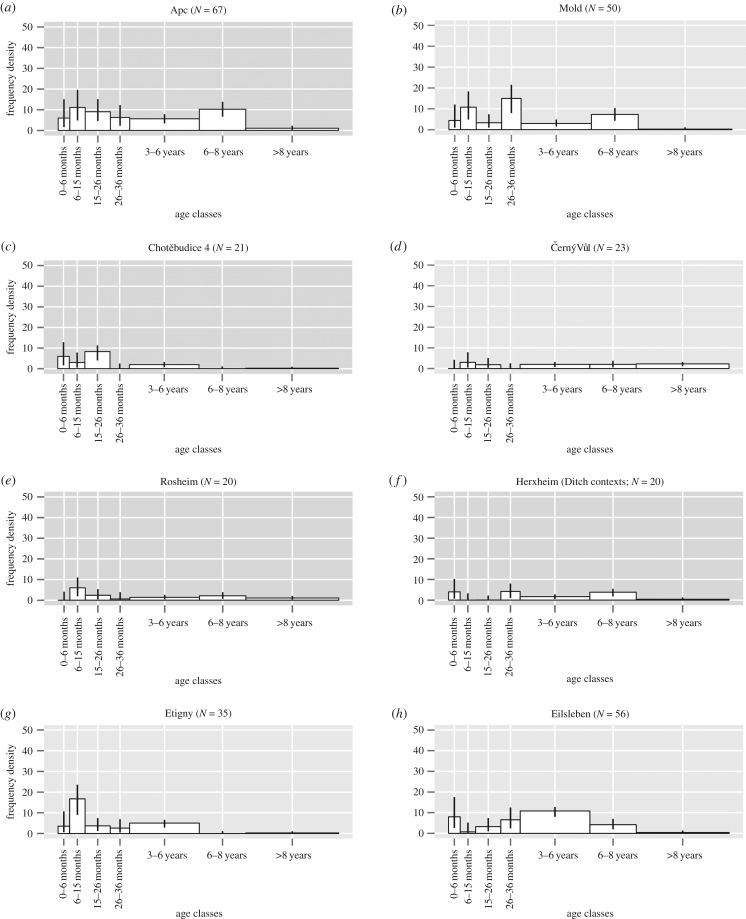
The mortality profiles from (*a*) Apc; (*b*) Mold; (*c*) Chotěbudice 4; (*d*) Černý Vůl; (*e*) Rosheim; (*f*) Herxheim (ditch contexts); (*g*) Etigny; (*h*) Eilsleben. Age classes follow Legge [35]. *N*, number of teeth.

The majority of the mortality profiles were characterized by high rates for age classes 6–15, 26–36 months and 6–8 years; however, there were exceptions (electronic supplementary material, S4). We chose eight of the 19 mortality profiles (figure 3) that clearly represent the overall trends that were evident in the profiles (figure 3*a,b*). These were chosen for two main reasons: good representative sample size and clearly illustrated trends such as the absence or evidence of slaughter in age classes 6–15 and 26–36 months, 3–6 and 6–8 years. There was no evidence for animals slaughtered in age class 26–36 months in the final phase of Chotěbudice, Černý Vůl and Rosheim (figure 3*c–e*), and no evidence of animals being slaughtered in age classes 6–15 months and 6–8 years at Herxheim (ditch contexts) and Etigny, respectively (figure 3*f*,*g*). There were some notable mortality profiles that seem to represent slaughter practices for specific modes of production such as in Eilsleben, where slaughter in age class 3–6 years was prominent (figure 3*h*). Overall, there was considerable similarity between all mortality profiles such as the consistent presence of teeth in age class 6–8 years, which suggests that similar types of slaughter management were practised at the sites of this study.

### (b) Correspondence analysis

CA is a descriptive statistical analysis and is used to highlight possible trends within the dataset. CA was based on 54 026 Dirichlet deviates generated from the age-at-death dataset. The overall inertia was 0.59 with 64.8% explained in the first three dimensions (F_1_: 24.2%: F_2_: 21.6%; F_3_: 18.7%). Table 1 presents the summary for the CA for the seven age classes. Based on the coordinates and contributions of individual age classes, we can determine what age classes explain the inertia for each axis. F_1_ was explained by the opposition between greater than 8 years, 6–15 months and 6–8 years, whereas F_2_ was by the opposition between 0 and 6 months, 26 and 36 months and 3 and 6 years. Finally, F_3_ was explained between greater than 8 years, 6–8 years and 15–26 months.

**Table 1.** Summary of the coordinates and contributions for age classes from CA. (Mass is equal to the marginal sum of the respective age class divided by the grand total of the table; axis coordinates (coord); axis contributions (Ctr), which is the contribution of each age class to the inertia of each axis [60]. The age classes with the highest Ctr are highlighted in grey for each axis.)

age class	mass	F_1_ coord	Ctr (‰)	F_2_ coord	Ctr (‰)	F_3_ coord	Ctr (‰)
0–6 months	100	0.308	67	−0.526	217	0.076	5
6–15 months	148	0.323	109	−0.078	7	−0.053	4
15–26 months	165	0.105	13	−0.157	32	−0.32	153
26–36 months	122	−0.155	21	−0.368	131	−0.198	44
3–6 years	214	−0.167	42	0.535	485	−0.262	133
6–8 years	151	−0.676	486	−0.116	16	0.458	287
greater than 8 years	100	0.611	263	0.376	112	0.642	374

**Figure 4.**
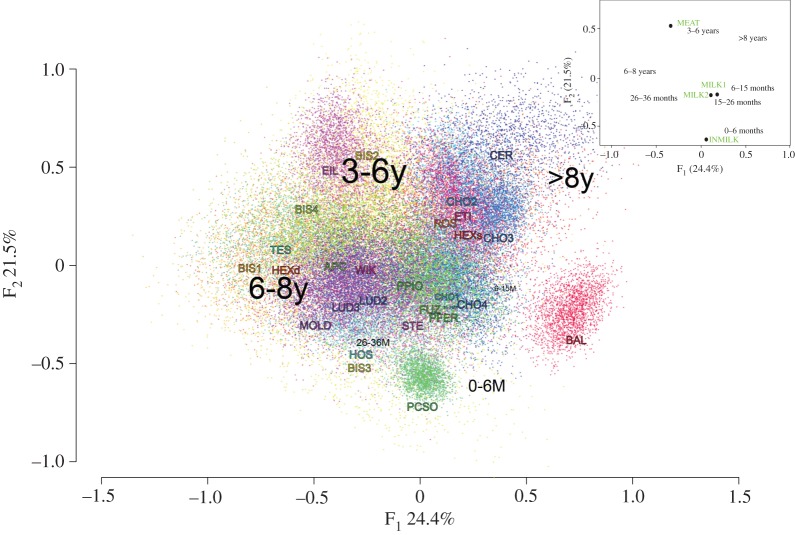
Correspondence analysis with Dirichlet deviates showing the F_1_ and F_2_ dimensions with each site represented by a different colour. The overall inertia is 0.59. The size of the age classes lettering reflects their contribution to each dimension [60]. The site codes are as follows: Apc-Berekalja (APC), Füzseabony-Gubakút (FUZ), Polgár-Piócási-dűlő (PPIO), Polgár-Ferenci-hát (PFER), Polgár-Csőszhalom-dűlő (PCSO), Tĕšetice-Kyjovice (TES), Hostivice-Sadová (HOS), Chotěbudice phase IIa (CHO1), Chotěbudice phase IIb (CHO2), Chotěbudice phase IIc-IIIa (CHO3), Chotěbudice phase IIIa–IIIb (CHO4), Černý Vůl (CER), Ludwinowo phase IIb (LUD1), Ludwinowo III (LUD2), Mold (MOLD), Eilsleben (EIL), Stephansposching (STE), Dillingen-Steinheim (WIK), Rosheim (ROS), Bischoffsheim (BIS1, 2, 3, 4), Herxheim-settlement (HEXs), Herxheim-ditch (HEXd), Etigny (ETI) and Balloy (BAL). (Inset) The CA of the original dataset (sites not shown) with the husbandry models (Meat, Milk1, 2 and Intense Milk) as supplementary points (open circles) and therefore do not contribute to the overall CA inertia.

We chose F_1_ and F_2_ to investigate further husbandry practices as they clearly differentiate: (i) the four reference profiles into two groups: milk and meat (figure 4, inset), and (ii) between groups of sites, which may be related to the practice of types of husbandry (figure 4, main). The post-lactation references positioned close to 6–15 and 15–26 months as expected. The meat reference profile was located close to the age class 3–6 years and the intense dairy reference plotted closely to zero to six months. The sites fall into two main groups: (i) a group closely associated with age class 6–8 years and to a lesser extent age classes 6–15, 15–26 and 26–36 months. This group contains the majority of the sites; and (ii) a group centred around age class 3–6 years and to a lesser extent greater than 8 years.

The post-lactation reference profiles fall within the first group. The group includes both phases of the site Ludwinowo where milk lipids were found in ceramic sieves. The presence of this site indicates a level of milk production was being practised at most LBK sites. Balloy plots away from the first group but was still closely associated with age class 6–15 months and therefore the post-lactation model profiles. The intense dairy management reference plots outside this group, which suggests that intensive dairy management was not common during the LBK. Polgar-Csőszhalom-dűlő was closely associated with age class zero to six months and consequently, the intense milk reference suggesting this strategy was practised at this site.

The meat reference profile falls within the second group centred on age class 3–6 years, which suggests that these herds were managed primarily for meat production. Age class greater than 8 years was also associated with this group, indicating older animals may also have been kept. All the sites associated with this group except for Eilsleben, Chotěbudice and Černý Vůl lie west of the Rhine. Eilsleben may also be different from the other sites as it was the northernmost site within the group. Overall, the CA suggests that cattle were managed similarly across the LBK cultural zone with a focus on maintaining animals until 6–8 years.

## 4. Discussion

The CA clearly delineates between reference profiles for post-lactation and intensive milk production, and meat exploitation. This result is strengthened by the fact that the post-lactation references (Bercy and Borduşani) are not only supported by their mortality profiles, but also by stable isotope analysis. The close correspondence between these reference profiles and the site of Ludwinowo where milk lipids were recovered strengthen the argument that the reference models do represent dairy management. The meat model is also unquestionable as it is based on an aurochs kill-off mortality profile. Only, the intensive dairy management model is more fragile, because it is not clear whether it results from intensive dairying based on young calf slaughtering or from perinatal mortality. Therefore, it is worthwhile to discuss this issue in more detail before a full discussion of the archaeological data.

### (a) Intensive slaughter model or high natural mortality?

Natural mortality in ruminants is highest within the first month. We recently demonstrated that perinatal mortality was highly variable in the Neolithic husbandry. At La Draga (Spain), greater than 50% mortality of animals in age class 0–1 months compared to 15% at Trasano (Italy) [57]. Refinement of mortality profiles suggests that infant mortality at Grimes Graves was the result of deliberate slaughter. Halstead [52] warned against the uniformity between prehistoric animals and modern breeds, i.e. the ability to let-down milk without influence of infants. The ‘post-lactation’ model provides a means of correcting for this issue, where infants are kept to ensure milk let-down. In an intensive model, cows may have been conditioned to let-down their milk using adoption or artificial methods [61]. It is clear from a review of ethnographic and modern examples from Europe and Turkey that there is no strict *uniformity* between modern cattle breeds [62]. Therefore, it is possible that let-down ability varied among Neolithic cattle and may even have varied between cattle lineages associated with the Danubian and Mediterranean routes. The positive effect of good husbandry skills, adequate fodder/forage and housing on milk let-down and yields indicates that the milking abilities of domesticates are not just controlled by genetics [52,63]. In the wild ungulate, lactation is naturally affected by similar external factors such as the availability of forage [63]. However, unless there is another proxy confirming milk exploitation such as organic residue analysis, we cannot be certain that the Grimes Grave profile reflects slaughter associated with dairy husbandry. Further analysis including other late Hungarian sites is needed to assess whether this type of slaughter was practised across the region.

### (b) Post-lactation slaughter model as mixed management

The post-lactation models chosen here also evidenced other practices such as meat exploitation and maintenance of mature animals. For this reason, post-lactation slaughter is commonly used by herders using traditional/unimproved specialized breeds such as those in Italy, France and Turkey [59,64,65] as a part of a dual system. Therefore, in such systems, calves—particularly male calves—are not slaughtered directly at the end of the lactation period, but some are chosen to mature for meat production. In addition, slaughtering could be timed with winter periods so as to reduce pressure on fodder provisions. It is important to consider that with every slaughter, the herd is diminished [33] and within closed systems, the rate of removal within a small herd has considerable impact in comparison to larger herds [29]. Herds managed of milk and mixed products would have been more viable than those that are managed exclusively for meat and can be a means of investing for the future [30]. The site of Balloy was positioned in the CA very close to the post-lactation model; this suggests a continuation of mixed husbandry approaches into subsequent cultural phases in the Paris Basin.

### (c) Specialization meat production

The use of an aurochs slaughter profile as representative of a pure meat subsistence strategy enables us to identify slaughter strategies focused on meat production. We are aware that the use of cattle tooth eruption, replacement and wear to age aurochs may potentially lead to misrepresentation of certain age classes. But given the close similarity between aurochs and cattle (despite different Latin names, biologically they are the same species), we can assume that the eruption and wear stages will be similar. Furthermore, slaughter between ages 3 and 6 years does not exclude maintaining mature animals or that the animals were managed for multi-purpose. This is evident from the close association between age class 3–6 years and the oldest age class. However, the results do suggest that there is a trend towards this type of slaughter at Černý Vůl, Chotěbudice (phase II and III), Bischoffsheim (phase II and IV), Rosheim, Eilsleben and Etigny. Given the size of a beef carcass at prime weight, it would have been necessary to share it among neighbours or to cure it for storage [29]. Providing fodder for a beast particularly male animals past economic necessity reflects conspicuous consumption. Meat in many societies past and present is only consumed during important ceremonies or after the natural death of an animal [31]. Communal feasting could have been seen as a means of maintaining togetherness of a community and could also have facilitated connection with neighbouring communities. It has been proposed that the best foods consumed at these events would have been the most labour intensive to produce. Therefore, this trend of specialized meat husbandry in the some regions of central Europe may be a reflection of raising prime cattle for meat production as a part of conspicuous consumption.

### (d) Linearbandkeramik cattle husbandry and landscape

The management of cattle herds would have been influenced by the stocking capacity of the landscape, i.e. the availability of water and sufficient fodder resources [66]. Woodland covered most of Europe during the Neolithic with the density and composition varying according to the underlying substrate and aspect. Natural openings would have been caused by lightning strikes and river courses, which would have allowed Neolithic farmers space to settle [67]. Maintaining pasture and winter fodder stocks for cattle and other livestock would have been practised throughout the year and possibly had an impact on the surrounding environment [66,68]. However, archaeological evidence is restricted to later prehistoric contexts in this region and to contemporary Mediterranean ones, and stable isotopic analysis of LBK cattle bones and teeth suggests little contribution of leaf fodder from dense forests [17,27,69–72]. Harvested fields would also have provided a source of fodder. Originally, it was proposed that arable crops would have been grown in shifting cultivation system; however, it is becoming more accepted that there would have been permanent fields within a woodland context [66,73]. From the composition of weed assemblages, it has been proposed that arable crops were planted in the spring, although these compositions can be affected by crop practices [66,74]. Sowing crops in spring would have reduced the risk of crop failure and provided additional grazing for domesticated animals during the autumn/winter, whereas the yield from winter-sown crops would have been greater owing to extra moisture from snow. In general, ruminants give birth when there is a guaranteed food supply, i.e. during the spring [75]. Therefore, in the latter scenario after harvest stubble crops could have been used as grazing for lactating cattle ensuring also the manuring of plots for the following growing season. Organization of these activities alongside agricultural practices would have formed the rhythm of LBK farmers' daily lives and shaped their annual cycle.

## 5. Conclusion

Linearbandkeramik cattle husbandry was previously believed to have been focused on meat production with some element of milk production. Our results show that slaughter management associated with dairy husbandry was practised in all LBK regions and periods as well as meat production. The detection of milk lipids in ceramics is clear proof dairying was practised. The level of milk production during the LBK was not intensive. Computational models are required to estimate the quantity of milk produced. The age class 6–8 years contributed most to the overall inertia, which suggests these individuals were present in most profiles and may reflect the presence of females slaughtered once they have stopped lactating. In comparison to caprines and pigs, cattle are slow to mature and so this accumulation and investment were a means to transfer assets between generations. Therefore, cattle played a dual role as both a consumable product and as an investment in a long-term strategy. The deliberate investment and maintenance of cattle would have increased the symbolic value of cattle. Milk production may have played an important role within the development of cattle symbolism. The results presented here in this respect are not conclusive and further organic residue analysis of LBK ceramics from multiple sites will help clarify the role and level of milk production in LBK societies.

Cattle played an deciding role in LBK societies and were not only used for their meat as previously suspected, but that most of the time they were managed for both milk and meat production. This dual role made it easier to adapt to different environments, and could have contributed to the rapid spread of the LBK culture from the Carpathian basin to Poland in the east, and the Paris basin in the west. The analysis did not evidence any marked difference between the cattle husbandry system across the LBK zone, but there were some indications of different subsistence focuses particularly in areas west of the Rhine. Furthermore, we also demonstrate that mature animals were maintained at most sites, which indicates that LBK farmers invested in their cattle herds. This would have required a certain level of organization of forage, housing and water supplies which alongside their agricultural activities would have shaped their annual cycles and reinforced the symbolic value of cattle that is seen during the Early and Middle Neolithic in central Europe.

## Supplementary Material

Mortality data based on dental eruption, replacement and wear stages.; R_code; Legge age classes for R code; Supplementary figures: Age-at-death profiles from studied sites

## Data accessibility

This article has no additional data.
