# Correction to ‘The evolution of dual meat and milk cattle husbandry in Linearbandkeramik societies’

**DOI:** 10.1098/rspb.2017.1748

**Published:** 2017-09-13

**Authors:** Rosalind E. Gillis, Lenka Kovačiková, Stéphanie Bréhard, Emilie Guthmann, Ivana Vostrovská, Hana Nohálová, Rose-Marie Arbogast, László Domboróczki, Joachim Pechtl, Alexandra Anders, Arkadiusz Marciniak, Anne Tresset, Jean-Denis Vigne

*Proc. R. Soc. B* **284**, 20170905 (Published Online 2 August 2017) (doi:10.1098/rspb.2017.0905)

An error caused by the lead author's oversight in a co-author's name and affiliation. It should be Alexandra Anders, Institute of Archaeological Sciences, Eötvös Loránd University, Múzeum krt. 4/B, 1088 Budapest, Hungary.

