# Supplementary material for: The evolution of dual meat and milk cattle husbandry in Linearbandkeramik societies
Source: Proc Biol Sci. 2017 Aug 2;284(1860):20170905. doi: 10.1098/rspb.2017.0905 (PMC5563807; doi:10.1098/rspb.2017.0905)
Supplement: Mortality data based on dental eruption, replacement and wear stages.; R_code; Legge age classes for R code; Supplementary figures: Age-at-death profiles from studied sites [file rspb20170905supp1.pdf]

ESM 1: Mortality data based on dental eruption, replacement and wear stages.

Site Name	Site code	Country	Climate*	Culture/ Date (cal. BC)	0-6M	6-15M	15-26M	26-36M	3-6Y	6-8Y	>8Y	Total N	Dating reference	Data reference
Apc-Berekalja	APC	Hungary	Dfb	TLP-LBK /(5470-4950)	3	7.4	9.8	5.2	16.9	20.5	4.3	67	Domboróczki et al. 2016, 2009	
Füzseabony -Gubakút	FUZ	Hungary	Dfb	ALP/ (5646–4910)	9.9	11.9	9.8	9.8	11.3	15.6	11.8	80	Whittle et al. 2013	
Polgár-Piócási-dűlő	PPIO	Hungary	Dfb	ALP/(5330-4940)	0	5.8	10	2.9	8.1	2.2	0	29		
Polgár-Ferenci-hát	PFER	Hungary	Dfb	ALP/ (5310-5060)	1.3	13.8	4.4	2.4	5.2	3.9	0	31		
Polgár-Csőszhalom-dűlő	PCSO	Hungary	Dfb	HLN/ (4840-4560)	20.7	18.4	34.1	31.8	10.9	10.3	0.8	127	Raczky et al. 2015	
Těšetice-Kyjovice	TES	Czech rep	Dfb	LBK Ib-IIa/ (5300-5200)	1.3	2.7	0.6	2.4	6.1	12	1.9	27	Pleinerová et al 2005; Řídký et al. 2007	
Hostivice-Sadová	HOS	Czech rep	Dfb	LBK/ (5450-5050)	3	5	14	2	2	13	2	41		Kovacikova unpub, Kovacikova et al. 2012
Chotěbudice	CHO1	Czech rep	Dfb	LBK IIa /(5300-5250	9	4	9	4	13	3	4	46		
Chotěbudice	CHO2	Czech rep	Dfb	LBK IIb/ (5400-5250	1	7	10	4	20	0	6	48		
Chotěbudice	CHO3	Czech rep	Dfb	LBK IIc-IIIa/ (5250-5100)	6	23	22	12	34	4	25	126		
Chotěbudice	CHO4	Czech rep	Dfb	LBK IIIa-IIIb/ (5250-5100)	3	2	9	0	6	0	1	21		
Černý Vůl	CER	Czech rep	Dfb	LBK IIc-IIIb/ (5200-5000)	0	2	2	0	6	4	9	23		
Ludwinowo	LUD2	Poland	Dfb	LBK IIB/ (5200-5100/5000)	1.9	4.4	12.1	3.1	8.2	8.6	1.8	40	Pyzel 2009	
Ludwinowo	LUD3	Poland	Dfb	LBK III/(5100/5000-4900)	8.7	10.6	10.9	12.3	20.9	19	0.6	83		
Mold	MOLD	Austria	Dfb	LBK Ib/ (5320–5070	2.2	7.2	3.6	12.5	8.9	14.6	1	50	Stadler et al. 1996	
Herxheim-settlement	HEX-set	Germany	Cfb	LBK final/ (5220-5020)	0.9	3.9	1.2	4.3	3.6	6.6	4.5	25	Denaire 2009	
Herxheim-ditch	HEX-dit	Germany	Cfb	LBK final / (5220-5020)	2	0	0.1	3.5	5	7.7	1.7	20		
Eilsleben	EIL	Germany	Cfb	LBK älteste/(5844-5472)	4	0.5	3.5	5.5	32.5	8.4	1.6	56	Hinz et al. 2012	Bréhard unpub
Stephansposching	STE	Germany	Cfb	LBK IIb-IIIc/ (5250-4900)	2	3.4	3.6	5.2	3.6	3.7	2.5	24	Pechtl unpub	
Dillingen-Steinheim	WIK	Germany	Cfb	LBK IIa-IIIb/ (5250-5100)	0	0	1.6	2	2.1	1.4	1	8		
Rosheim	ROS	France	Cfb	Rubane recent/(5224-4954	0	4	2.6	0.5	4.2	4.3	4.4	20	Denaire et al. Unpub	
Bischoffsheim	BIS1	France	Cfb	Rubane ancient/(5328-5055	0	0	1	3	3	6	1	14		
Bischoffsheim	BIS2	France	Cfb	Rubane moyen/(5467-5011)	0	0	3	0.7	6.9	1	0.4	12		
Bischoffsheim	BIS3	France	Cfb	Rubane moyen/IV/(5432-5008)	4	0	0	0	1	4	1	10		
Bischoffsheim	BIS4	France	Cfb	Rubane IV/ (5300-5060)	0	1	1	0.4	4	5.1	1.5	13		
Etigny	ETI	France	Cfb	RRBP/(4834 to 4800	1.8	11.2	4	2.2	15	0	1	35	Dubouloz 2003	Carré 2004
Balloy	BAL	France	Cfb	VSG/ (4714-4556)	16.5	17.9	5.1	3.8	3	2.5	17	66		Tresset 1996
**Mortality profile references**														
Popină Bordușani[1]	Milk 1	Romania	Post-lactation	Chalcolithic: late 5th millennium BC	11.3	30.7	35	25.8	23.7	15.7	15.8	158		after Bréhard and Bălăşescu 2012
Bercy[2]	Milk 2	France	Post-lactation	Chasseen: late 5th millennium BC	24	23.65	46.83	57.5	53	9	17	231		Tresset 1996
Grimes Grave [3]	Intense Milk	United Kingdom	Intensive Milk	Bronze Age	84	36	17	42	28	38	9	254		Legge 1992
La Montagne[4]	Meat	France	Meat	Mesolithic	0	2.2	3.9	5.4	19.3	6.2	2	39		Helmer and Monchot 2006

*Peel et al. 2007, Dfb: Cold with no dry season and warm summer, Cfb: Temperate with no dry season and warm summer.

Biblography

Bréhard S. and Bălăşescu A. 2012. What's behind the tell phenomenon? An archaeozoological approach of Eneolithic sites in Romania? *Journal of Archaeological Science* **39**(10): 3167-3183.

Denaire A. 2009. Radiocarbon dating of the western European Neolithic: Comparison of the dates on bones and dates on charcoals *Radiocarbon* 51(2): 657-674.

Domboróczki L., Budek, A., Daróczi-Szabó L., Kaczanowska M., Kalicki R., Kłusakiewicz E., Kozłowski J.K. Kreuz A., Pomázi P., Wasilewski M., Zoffmann K.Zs. 2016. Excavation along the easternmost frontier of the LBK in NE Hungary at Apc-Berekalja I. (2008-2009). Archaeologiai Értesítő 141: 1-27.

Domboróczki L. 2009. Settlement structures of the Alföld Linear Pottery Culture (ALPC) in Heves County (North-Eastern Hungary): development models and historical reconstructions on mico, mezo and macro levels *In*: J.K. Kozłowski (ed.) *The Interations between different models of Neolithization North of the Central European Agro-Ecological Barrier* Polska Akademia Umiejetności, Prace Komisji Prehistorii Karpat, 4. Kraków : 75-127.

Dubouloz J., 2003. Datation absolue du premier Néolithique du Bassin parisien: complément et relecture des données RRBP et VSG *Bulletin de la Société* **100** (4): 671-689.

Helmer D. and Monchot, H. 2006. Un site mésolithique de chasse à l'aurochs (La Montagne, Sénas, Bouches-du-Rhône). *Anthropozoologica* 41 (2): 215-228.

Hinz M., Furholt M., Müller J., Raetzel-Fabian D., Rinne C., Sjögren K.-G., Wotzka H.-P. 2012. RADON - Radiocarbon dates online 2012. Central European database of ^{14}C dates for the Neolithic and Early Bronze Age. *www.jungsteinsite.de* . 1-4.

Kovačiková L., Bréhard S., Šumberová R. Balaase M. and Tresset A. 2012. The new insights into the subsistence and early farming from Neolithic settlements in Central Europe: the archaeozoological evidence from the Czech Republic. *Archaeofauna* **21**: 71-97

Legge A.J. 1992. *Excavations at Grimes Graves Norfolk 1972-1976 Fascicule 4: Animals, Environment and the Bronze Age Economy.* London, British Museum Press.

Pleinerová I., Klementová J., Hložek J., Daněček D. 2005. Záchranný archeologický výzkum v lokalitě Sadová I.+II., Hostivice, k.ú. Hostivice. Zprávy České archeologické společnosti.

Pyzel, J. 2009. Setlement history of the Linear Band Potery culture in Kuyavia. In: D. Hoffman and P. Bickle (eds.) *Creating Communities New Advances in Central European Neolithic Research* . Oxford: Oxbow books. Pp. 71-79.

Peel M. C., Finlayson B. L. and Mahon T. A. 2007. Updated world map of the Köppen-Geiger climate classification. *Hydrological Earth Systems Science* **11**, 1633–1644.

Raczky P., Anders A., Sebők K., Csippán P. and Tóth Zs. 2015. The times of Polgár-Csőszhalom. Chronologies of human activities on the Polgár-Csőszhalom horizontal settlement. In: Hansen, S.–Raczky, P.–Anders, A.–Reingruber, A. (eds): *Neolithic and Copper Age between the Carpathians and the Aegean Sea. Chronologies and Technologies from the 6th to the 4th Millennium BCE. International Workshop Budapest 2012.* Archäologie in Eurasien 31, Bonn 2015, 21–48.

Řídký J., Stolz D., Zápotocká M. 2007. Neolithic settlement in Černý Vůl (Prague-west). Forms of features, pottery analysis and chipped stone industry from excavations in 1975-77 and 1914. Praehistorica 28: 177-236.

Stadler P., Lenneis E. and Windl H., 1996. Neue 14C-Daten zum Frühneolithikum in Österreich. *Prehistoire Européenne* **8**: 97-116.

Tresset A. 1996. Le rôle des relations homme-animal dans l'évolution économique et culturelle des sociétés des Vème-VIème millénaires en Bassin Parisien. Paris, Université de Paris I, Panthéon-Sorbonne.

Supplementary data2: R_code

```
library("ggplot2")       # to plot histograms with time correction

library("grid")          # to plot histograms with grid.arrange()

library("gridExtra")   # to plot histograms with grid.arrange()
library("gtools")        # to use rdirichlet()

library("ca")            # to use ca() for correspondence analysis; version 0.55

library(LaplacesDemon)

library(MASS)           # kde2d density estimation

dirch_prior=0.5

nsim=2000

Legge_class_2 <- ESM3

View(Legge_class_2)
legge_class=Legge_class_2[,2]

# Time correction in years:
time_correc = legge_class/12

# Define the age class columns containing the minimum
# number of individuals in each age class
ageClass_col = c(2:8)

# total number of age classes
nbClasses=length(ageClass_col)

# SDATA1 without foreign symbols, climate, references. Headers are Site code; Age classes (2:9) and removing the models from the analysis.
data <- ESM1

nbSites=length(data[,1])

SiteCode=data[,1]

# GRAPHS with credible intervals using Dirichlet
{

  plots = list()   # new empty list

for (i in 1:nbSites){

ageProfileMAT = matrix(data=as.numeric(as.matrix(data[i,ageClass_col])), nrow=nbClasses, ncol=1, byrow=TRUE)
ageProfileDFcorrec = as.data.frame(ageProfileMAT
colnames(ageProfileDFcorrec) = "site"
ageProfileDFcorrec$x = colnames(data)[ageClass_col]

ageProfileDFcorrec$width = time_correc
ageProfileDFcorrec$w = cumsum(ageProfileDFcorrec$width)

ageProfileDFcorrec$wm = ageProfileDFcorrec$w - ageProfileDFcorrec$width
ageProfileDFcorrec$wt = with(ageProfileDFcorrec, wm + (w - wm)/2)

 # the middle x coordinate of each age class
###--- 1.
# An empty Dirichlet matrix to record the simulated dataset
```

```
Dirichlet_simmat = NULL
Dirichlet_simmat = rbind(Dirichlet_simmat, rdirichlet(nsim, dirch_prior+t(ageProfileMAT)))
Dirichlet_simobs = rbind(Dirichlet_simmat, t(ageProfileMAT)/sum(t(ageProfileMAT)))
Dirichlet_simobs = Dirichlet_simobs*sum(t(ageProfileMAT))

# tail(Dirichlet_simobs) => the last row should be EXACTLY
# the observed...
###--- 2.
# Now, adjust for time correction and check with the observed
# (last row) that it works:
Dirichlet_simobscorrected = NULL
for (s in c(1:length(Dirichlet_simobs[,1]))){
  Dirichlet_simobscorrected = rbind(Dirichlet_simobscorrected, Dirichlet_simobs[s,]/time_correc)
}
###--- 3.
# Compute 95% credibility interval
CI95 = p.interval(Dirichlet_simobscorrected, HPD=FALSE, MM=FALSE, plot=FALSE)

###--- 4.
# Plot histogram overlaid with 95% CI
# to get histogram on 1 y scale:
DIRybounds = c(0, (max(Dirichlet_simobscorrected)+1))

# This is the common histogram
aP_correcH <- ggplot(ageProfileDFcorrec, aes(ymin = DIRybounds[1])) + theme(legend.position="none") +
ggtitle(paste(SiteCode[i], ', N of teeth=', round(sum(data[i,ageClass_col])), sep='')) + ylim(0, 50) +
geom_rect(mapping=aes(xmin = wm, xmax = w, ymax = site*1/width, fill = x), size=0.3, fill="white", color="black",
alpha=1) + xlab("age classes") + ylab("Frequency density") + scale_x_continuous(breaks=ageProfileDFcorrec$wt,
labels=ageProfileDFcorrec$x) + theme(panel.grid.minor=element_blank())



# overlay with 95% CI:
aP_correcHi <- aP_correcH +
  # Plot the vertical lines of the 95% CI at the
  # midpoints: ageProfileDFcorrec$wt
  geom_segment(x=ageProfileDFcorrec$wt, y=CI95[,i:nbSites], xend=ageProfileDFcorrec$wt, yend=CI95[,2])


# add aP_correcHi to plot list
plots[[i]] <- aP_correcHi
}
}

grid.arrange(plots[[1]], plots[[2]])#repeat if necessary


##################CA analysis

ageProfileMAT = matrix(data=as.numeric(as.matrix(data[,ageClass_col])), nrow=nbSites, ncol=nbClasses, byrow=FALSE)
colnames(ageProfileMAT) = colnames(data)[ageClass_col]
row.names(ageProfileMAT) = SiteCode

uncorrected_simmat = NULL
maxSS = 0
for (i in 1:nbSites){
     uncorrected_simmat = rbind(uncorrected_simmat, rdirichlet(nsim, dirch_prior+ageProfileMAT[i,])*sum(ageProfileMAT[i,]))
     if (sum(ageProfileMAT[i,])>maxSS){maxSS=sum(ageProfileMAT[i,])}
 }

null_simmat = rdirichlet(nsim, rep(dirch_prior, nbClasses))*maxSS
rindex = seq(1, nrow(uncorrected_simmat))
uncorrected_rsimmat = uncorrected_simmat[rindex,]

# Append null_simmat to uncorrected_rsimmat
```

```
uncorrected_rsimmat = rbind(uncorrected_rsimmat, null_simmat)

# and then attach observed data as the last rows
uncorrected_rsimmat = rbind(uncorrected_rsimmat, ageProfileMAT)

# CA of simulated data
SIM = ca(uncorrected_rsimmat)

#extracting the xy coordinates
x=summary(SIM)

rows=cbind(x$rows[,5],x$rows[,8])
cols=cbind(x$columns[,5],x$columns[,8])

rows_cor=rows/1000
col_cor=cols/1000

#extracting the contributions to the axis made by the age classes.
ctr12=x$columns[1:7,7]+x$columns[1:7,10]
ctr12_cor=ctr12/1000

# Graphical limits
DIRxmin = min(min(rows_cor[,1:2]), min(cols_cor[,1:2]))
DIRxmax = max(max(rows_cor[,1:2]), max(cols_cor[,1:2]))
DIRymin = DIRxmin
DIRymax = DIRxmax

#delimination of what to plot
null_from = nsim*nbSites + 1
null_to = nsim*nbSites+nsim
colrNO = seq(1, nbSites, length=nbSites)
colrs=sort(rep(colrNO, nsim))

##plot of ca Dirichlet simulated data


plot(rows_cor[(null_from:null_to),1:2], xlim=c(DIRxmin, DIRxmax), ylim=c(DIRymin, DIRymax), xlab=paste("F1 ",
round(summary(uncorrected_ca)$scree[1,3], digits=1), "%", sep=''), ylab=paste("F2 ",
round(summary(uncorrected_ca)$scree[2,3], digits=1), "%", sep=''), col= "grey", pch=20, cex=0.18)
text(x=rows_cor[(length(rows_cor[,1])-nbSites+1):length(rows_cor[,1]),1], y=rows_cor[(length(rows_cor[,2])-
nbSites+1):length(rows_cor[,2]),2], col=rainbow(nbSites)[colrNO], SiteCode, pch=4, cex=1)


#To add age classes adjusted to reflect the age class contribution to each axis

text(col_cor[,1], col_cor[,2], colnames(ageProfileMAT), pch=20, col="black", cex=5*ctr12_cor)
```

Supplementary data3: Legge age classes

Ageclass	Months
0-6M	6
6-15M	8
15-26M	13
26-36M	10
3-6Y	36
6-8Y	24
8-10Y	24
>10Y	24

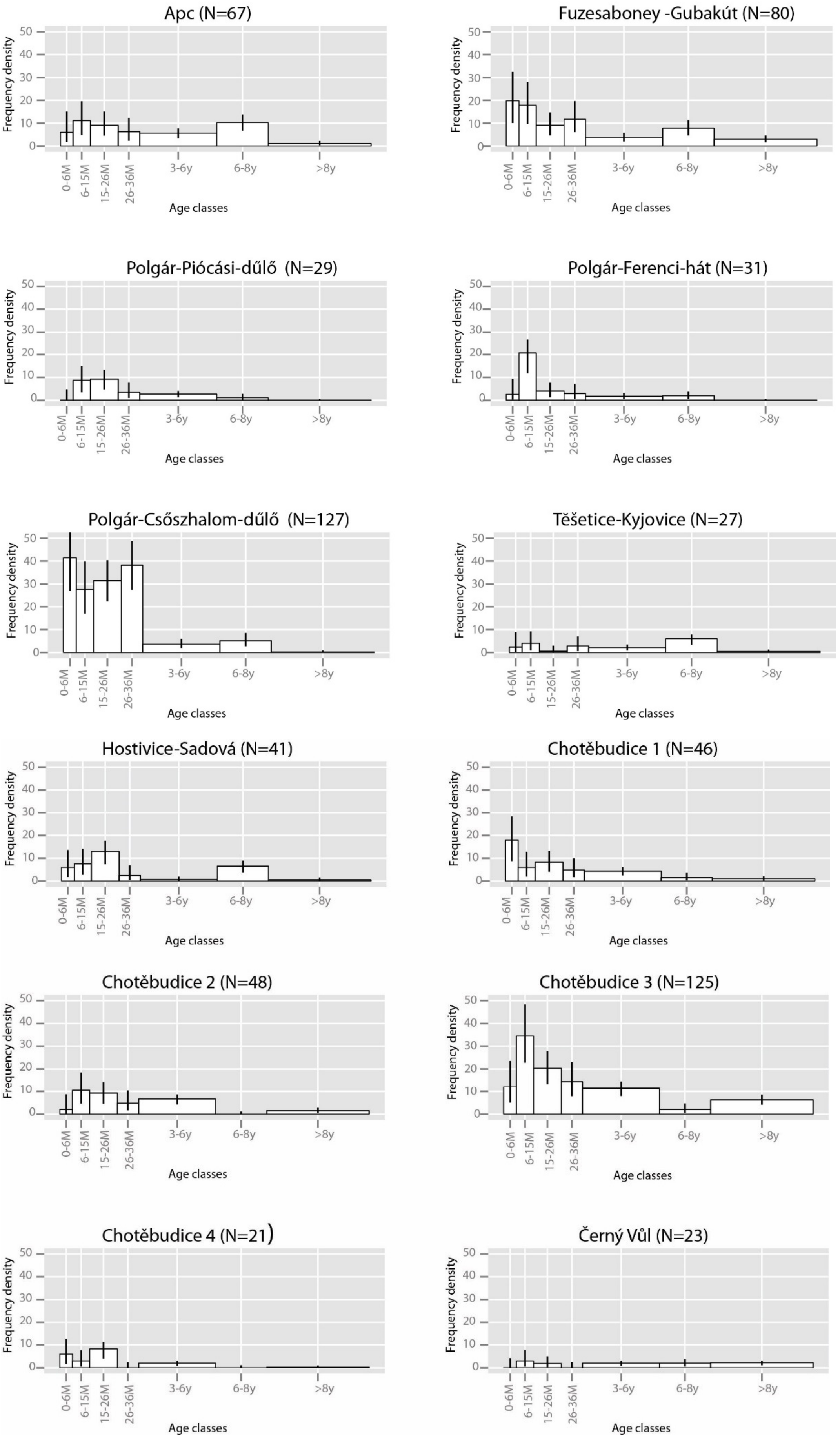

Apc (N=67)
Fuzesaboney -Gubakút (N=80)
Polgár-Piócási-dűlő (N=29)
Polgár-Ferenci-hát (N=31)
Polgár-Csőszhalom-dűlő (N=127)
Těšetice-Kyjovice (N=27)
Hostivice-Sadová (N=41)
Chotěbudice 1 (N=46)
Chotěbudice 2 (N=48)
Chotěbudice 3 (N=125)
Chotěbudice 4 (N=21)
Černý Vůl (N=23)
Frequency density
0
10
20
30
40
50
0-6M
6-15M
15-26M
26-36M
3-6y
6-8y
>8y
Age classes

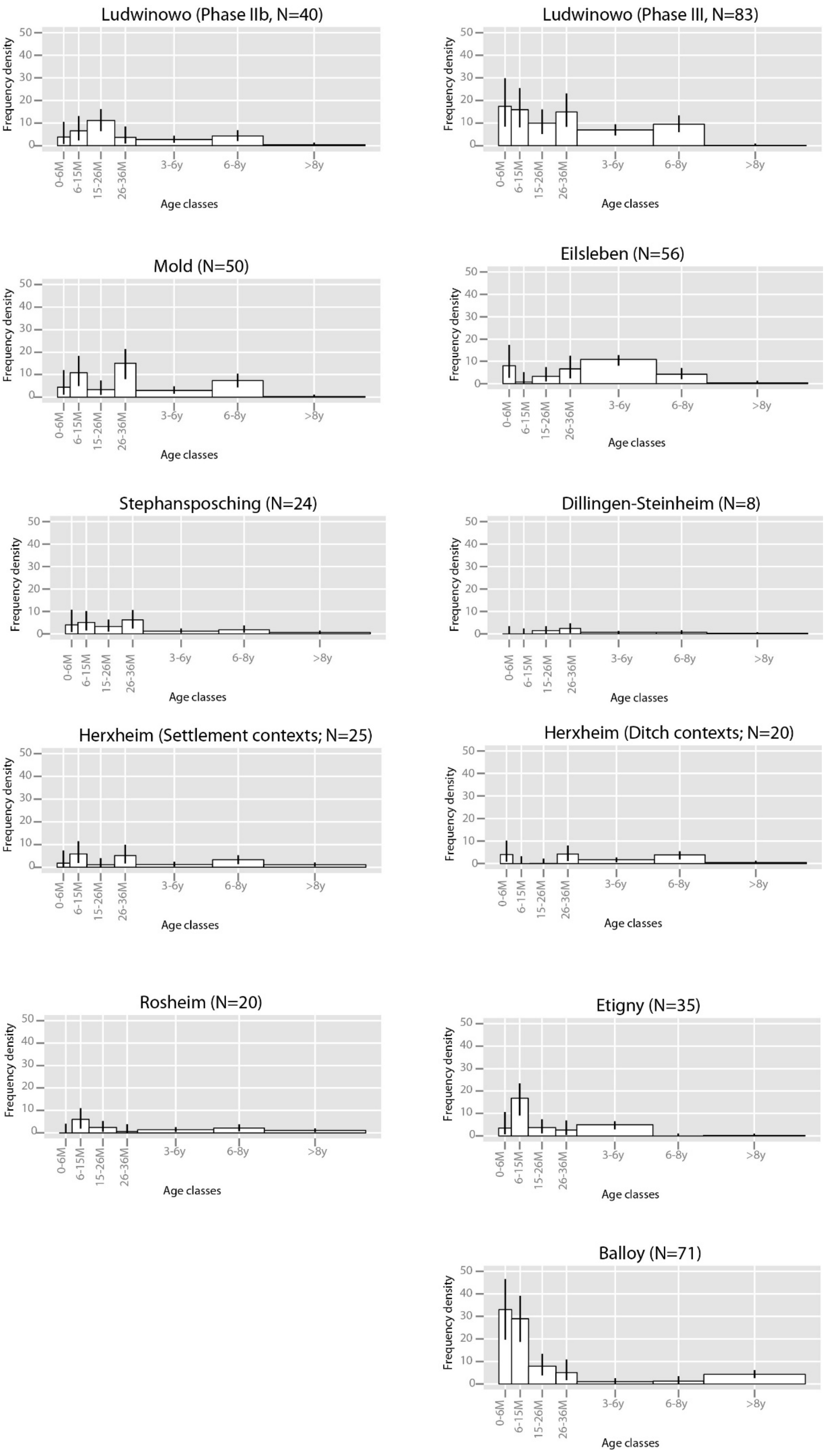

Ludwinowo (Phase IIb, N=40)
Ludwinowo (Phase III, N=83)
Mold (N=50)
Eilsleben (N=56)
Stephansposching (N=24)
Dillingen-Steinheim (N=8)
Herxheim (Settlement contexts; N=25)
Herxheim (Ditch contexts; N=20)
Rosheim (N=20)
Etigny (N=35)
Balloy (N=71)
Frequency density
0
10
20
30
40
50
0-6M
6-15M
15-26M
26-36M
3-6y
6-8y
>8y
Age classes

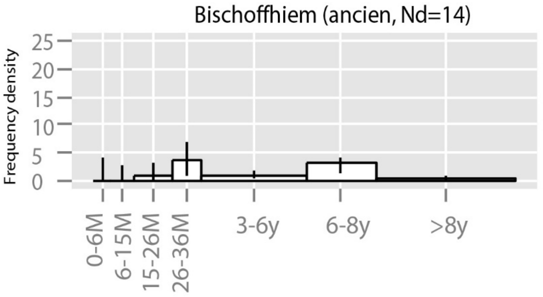

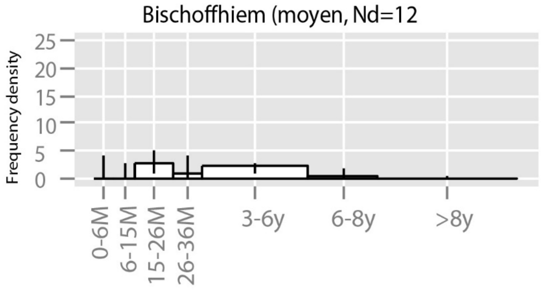

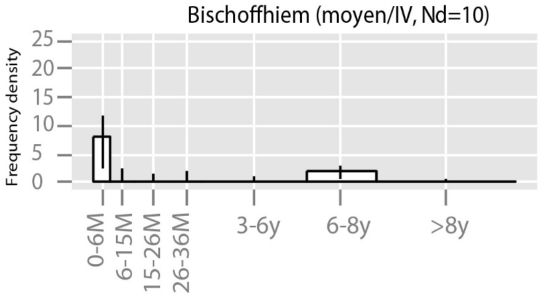

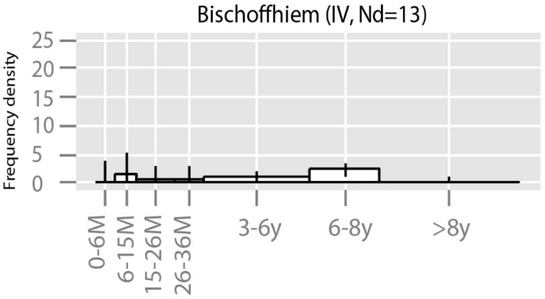

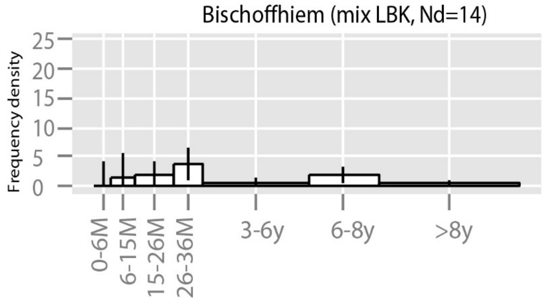

Supplementary figures: Mortality profiles from Apc-Berekalja, Füzseabony-Gubakút, Polgár-Piócási-dűlő, Polgár-Ferenci-hát, Polgár-Csőszhalom-dűlő (Hungary), Hostivice-Sadová, Chotěbudice, Černý Vůl, Těšetice-Kyjovice(Czech republic), Ludwinowo (Poland), Mold, Elisleben, Stephansposching, Dillingen-Steinheim, Herxheim (Germany), Rosheim, Balloy and Bischoffsheim (France).
